# LINC02126 is a potential diagnostic, prognostic and immunotherapeutic target for lung adenocarcinoma

**DOI:** 10.1186/s12890-022-02215-4

**Published:** 2022-11-10

**Authors:** Junbin Wang, Jixian Liu, Qinghua Hou, Mengmeng Xu

**Affiliations:** 1 Department of Thoracic Surgery, Peking University Shenzhen Hospital, No. 1120 Lianhua Road, Futian District, Shenzhen, 518034 Guangdong China; 2 HaploX Biotechnology, Shenzhen, China

**Keywords:** Lung adenocarcinoma, LINC02126, Immune cells, Diagnosis, Prognosis, Tumor mutation burden

## Abstract

**Background:**

Adenocarcinoma has long been an independent histological class of lung cancer, which leads to high morbidity and mortality. We aimed to investigate the contribution of LINC02126 in lung adenocarcinoma.

**Methods:**

RNA sequencing data and clinical information were downloaded. Diagnostic efficiency and survival analysis of LINC02126 were performed, followed by functional analysis of genes co-expressed with LINC02126 and differentially expressed genes (DEGs) in different LINC02126 expression groups. Tumor immune microenvironment (TIME) cell infiltration and correlation analysis of tumor mutation burden were performed in different LINC02126 expression groups.

**Results:**

In lung adenocarcinoma, the expression level of LINC02126 was significantly decreased. Significant expression differences of LINC02126 were found in some clinical variables, including T staging, M staging, sex, stage, and EGFR mutation. LINC02126 had potential diagnostic and prognostic value for patients. In the low LINC02126 expression group, the infiltration degree of most immune cells was significantly lower than that in the high LINC02126 expression group. Tumor mutation burden level and frequency of somatic mutation in patients with low LINC02126 expression group were significantly higher than in patients with high LINC02126 expression group.

**Conclusions:**

LINC02126 could be considered as a diagnostic, prognostic and immunotherapeutic target for lung adenocarcinoma.

**Supplementary Information:**

The online version contains supplementary material available at 10.1186/s12890-022-02215-4.

## Background

Lung cancer (LC) is the malignant tumor with the highest morbidity and mortality in the world [1]. Lung adenocarcinoma accounts for approximately 40% of all LC and results in more than 500,000 deaths each year [2]. The diagnosis of lung adenocarcinoma remains poor. Most patients with lung adenocarcinoma are already at advanced or metastatic stages when first diagnosed [3, 4]. In addition, the prognosis for lung adenocarcinoma is poor. Histopathological and sub-classification of lung adenocarcinoma is unclear and it tends to metastasize at the early stage of the disease [5, 6]. High rates of recurrence and distant metastasis of lung adenocarcinoma have made it a major threat to patient health [7–11]. The traditional treatment for advanced lung adenocarcinoma is surgery, chemotherapy and radiotherapy, but the therapeutic effect is not satisfactory. Therefore, it is urgent to explore the underlying tumorigenesis and progression for insights into the identification of potential markers, which may guide the development of new diagnostic strategies and targeted therapies.

With further study of tumors, researchers have found some abnormal expression of long non-coding RNAs (lncRNAs) mainly expressed in tumor tissues. LncRNA is a regulatory non-coding RNA with more than 200 nucleotides, which have a variety of gene expression regulation modes. Although the functions of most lncRNAs have not been characterized, they play an important role in regulating cancer metastasis [12–17]. In addition, lncRNAs acts as potential biomarkers that have predictive value for the survival of patients with cancer. Prostate cancer antigen 3 (PCA3) is regarded as a key biomarker in prostate cancer [18, 19]. Metastasis-associated lung adenocarcinoma transcript 1 (MALAT1) and colon cancer-associated transcript 2 (CCAT2) are acted as biomarkers in patients with lung cancer [20–22]. The expression of LOC285548 and DKFZP434 L187 is negatively related to the overall survival of lung adenocarcinoma patients [23]. However, there is no report about LINC02126 in the development in lung adenocarcinoma. In view of this, we aimed to investigate the expression and function of LINC02126 in lung adenocarcinoma patients and its significance in early diagnosis and prognosis of lung adenocarcinoma.

## Materials and methods

### Dataset filtering and data preprocessing

The common gene expression data were retrieved from the Cancer Genome Atlas (TCGA) dataset. For the lung adenocarcinoma dataset, RNA sequencing data and clinical information were downloaded from the University of California, Santa Cruz (UCSC) Xena (https://gdc.xenahubs.net), which involving 526 lung adenocarcinoma tissue samples and 59 adjacent tissue samples. Detailed process of data preprocessing is as follows: (1) Those samples with unknown survival time, survival time less than 0 day, and without clinical follow-up information and survival status were removed; (2) The probe was converted to the gene symbol; (3) Probes corresponding to multiple genes were removed; (4) For multiple probes corresponding to a gene, the average was taken. After data preprocessing, 497 lung adenocarcinoma tissue samples and 59 adjacent tissue samples were used for further analysis. In addition, after screening and comparison in the TCGA and Genotype-Tissue Expression (GTEx) dataset, LINC02126 was found to be closely related to lung adenocarcinoma. Therefore, LINC02126 was the research focus of this study.

### Diagnostic efficiency analysis of LINC02126

Differential expression analysis of LINC02126 between lung adenocarcinoma and adjacent tissues was analyzed by Wilcon.text. To investigate the role of LINC02126 in lung adenocarcinoma patients, T test was utilized to analyze the expression of LINC02126 in different clinical indicators including age, sex, stage, and EGFR mutation. The predictive receiver operating characteristic (pROC) package in R language was utilized to analyze the diagnostic value of LINC02126. The area under the curve (AUC) represents the diagnostic capability of LINC02126.

### Survival analysis of LINC02126

In this study, lung adenocarcinoma patients were divided into high expression and low expression groups based on the median expression value of LINC02126. Kaplan–Meier, log-rank tests, univariate and multivariate Cox proportional risk regression models were used to compare the prognostic differences. A time-dependent ROC curve was applied to assess the accuracy of LINC02126 expression in predicting prognosis for lung adenocarcinoma. On the R platform, the contribution of LINC02126 expression to the prognosis of lung adenocarcinoma was evaluated by the RMS package. Combined effect survival analysis was used to evaluate the prognostic value of LINC02126 expression combined with clinical parameters for lung adenocarcinoma.

### Estimate of tumor immune microenvironment (TIME) cell infiltration

The single sample gene set enrichment analysis (ssGSEA) algorithm was used to quantify the relative abundance of each cell infiltration in the TIME of lung adenocarcinoma patients. The gene set was obtained from the previous study, which was rich in a variety of human immune cell subtypes. Enrichment score was used to represent the relative abundance of infiltrating cells at each TIME in each sample. The immune score, stromal score, tumor purity, and ESTIMATE score was calculated for each lung adenocarcinoma patient using the ESTIMATE algorithm through the “ESTIMATE” package in R. Wilcoxon.test was applied to compare the differences in immune cell infiltration, immune score, stromal score, tumor purity and ESTIMATE score in different LINC02126 expression groups.

### Correlation analysis between LINC02126 and tumor mutation burden

Mutation data of lung adenocarcinoma patients were downloaded from TCGA data set. Spearman correlation coefficient was utilized to evaluate the correlation between LINC02126 and tumor mutation burden, and further evaluate the possibility of immunocheckpoint inhibitor treatment in different LINC02126 expression groups.

### Functional analysis of genes co-expressed with LINC02126

The pearson correlation coefficient was used to identify genes co-expressed with LINC02126. *P* < 0.05 and |cor|> 0.3 was the threshold value to identify LINC02126-gene relational pairs of co-expression. The functional evaluation of LINC02126-co-expressed gene relational pairs was performed through annotation, visualization, and Integrated Discovery (DAVID) database. *P* < 0.05 was considered statistically significant.

### Functional and protein–protein interaction (PPI) analysis of differentially expressed genes (DEGs) in different LINC02126 expression groups

To reveal the prognostic mechanism between different LINC02126 expression levels, "limma" package was used to screen DEGs between different LINC02126 expression groups in tumor tissues of lung adenocarcinoma patients. |log_2_fold change (FC)|≥ 0.585 and false discovery rate (FDR) < 0.05 was the screening criteria of DEGs in high and low LINC02126 express groups. The DAVID database was used to identify biological functions of these DEGs. *P* < 0.05 was considered statistically significant. In addition, PPI network was constructed by online tool of STRING to analyze the interactions between these DEGs in lung adenocarcinoma. In addition, the regulatory relationship between LINC02126, miRNAs and common genes (between DEGs and co-expressed genes with LINC02126) was analyzed. Firstly, based on miRWalk database (http://mirwalk.umm.uni-heidelberg.de/), miRNAs interacting with common genes were identified under the screening criteria of “binding = 1”. Secondly, GSE74190 dataset (miRNA expression dataset) was downloaded from the Gene Expression Omnibus (GEO) database (https://www.ncbi.nlm.nih.gov/geo/), which contains 36 lung adenocarcinoma tumor samples and 44 adjacent normal tissue samples. The “llimma” package in R was used to identify differentially expressed miRNAs under the screening threshold of *P* < 0.05. The common miRNAs, identified in miRWalk database (negatively regulated common genes) and GSE74190 dataset, were identified.

### Statistical analysis

Statistical analysis was performed using R version 3.5.3. Wilcoxn.test was used to analyze expression levels of LINC02126 in lung adenocarcinoma. The survival curve was generated by Kaplan–Meier method. The difference between two groups was compared by log rank test. Univariate and multivariate analyses were performed by the Cox regression model. The independent prognostic value of LINC02126 was determined in combination with other clinical features. ROC curves were used to estimate the predictive efficiency of risk models for 1, 3, and 5 years of overall survival. *P* < 0.05 was considered statistically significant.

## Results

### Expression and diagnostic analysis of LINC02126

**Fig. 1:**
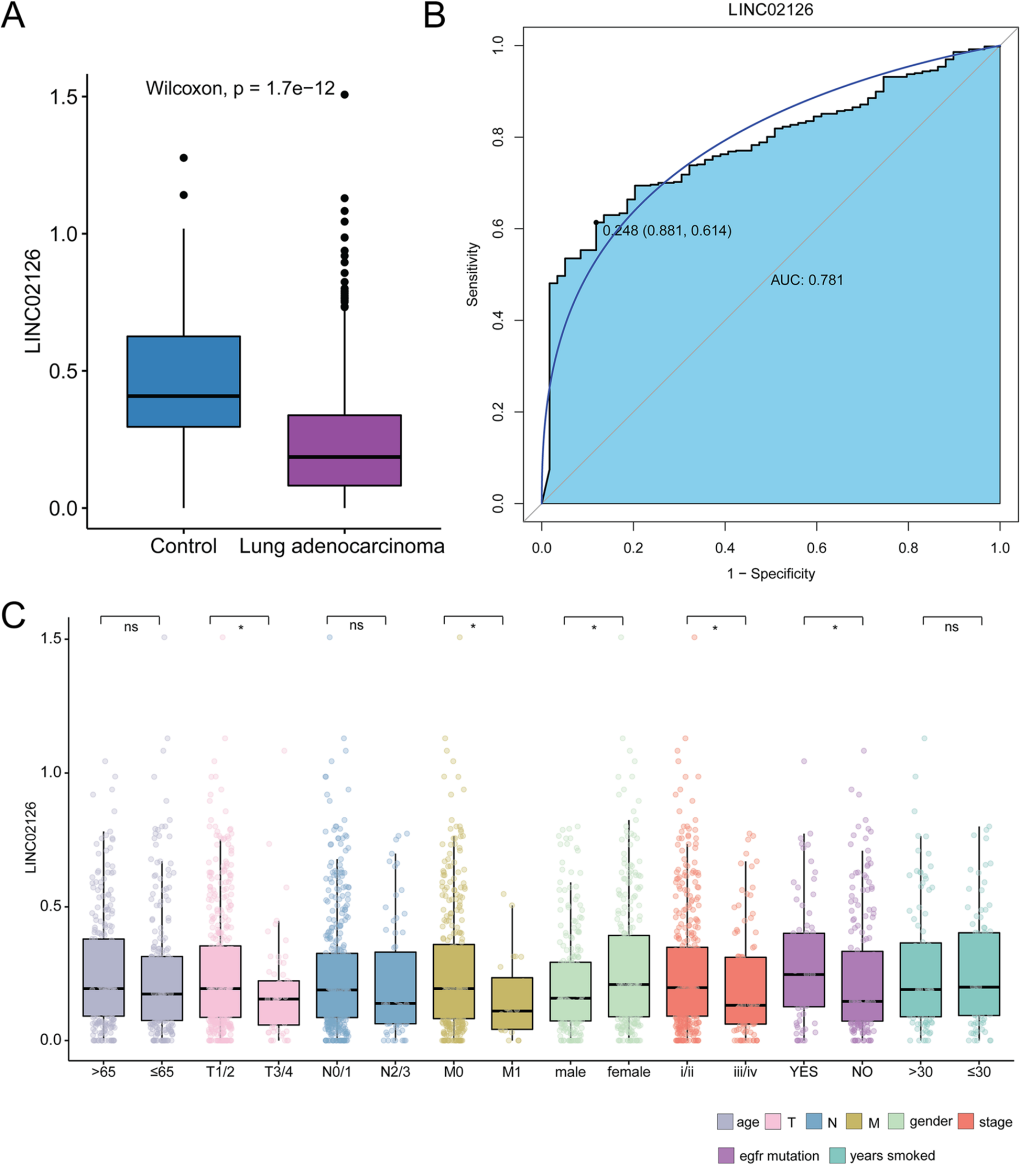
**A** Expression of LINC02126 in tumor tissue of lung adenocarcinoma Control is adjacent normal. **B** Diagnostic analysis of LINC02126 in lung adenocarcinoma. **C** Expression of LINC02126 in different clinical indicators of lung adenocarcinoma. *P < 0.05; NS: not significant

In lung adenocarcinoma, the expression of LINC02126 was significantly lower than that in adjacent tissues (Fig. 1A). Moreover, LINC02126 had a potential diagnostic value (AUC = 0.781) for lung adenocarcinoma (Fig. 1B). In addition, the expression of LINC02126 was analyzed in different clinical variables (Fig. 1C). The result showed that significant expression differences of LINC02126 were found in T staging (low expression in T3/4), M staging (low expression in M1), sex (low expression in male), stage (low expression in iii/iv), EGFR mutation (high expression in mutation). The above results indicated that LINC02126 expression was significantly abnormal and had a high accuracy in the disease diagnosis, which could be used as a potential diagnostic biomarker of lung adenocarcinoma.

### Survival analysis of LINC02126

**Fig. 2:**
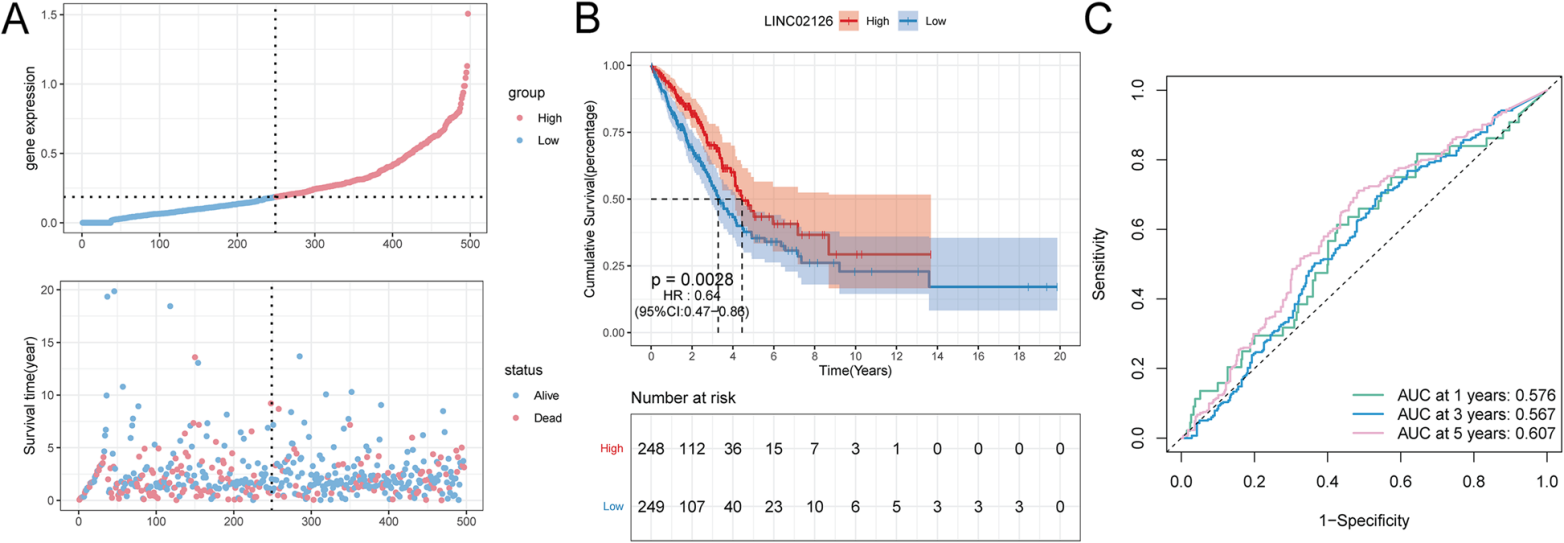
Survival analysis of LINC02126 in lung adenocarcinoma **A** Distribution of LINC02126 expression and survival status; the X axis represents the sample order; **B** Survival curves between high and low LINC02126 expression groups; numbers at risk indicates the number of individuals alive at the above time point (**C**) LINC02126 expression predicts the time-dependent ROC curve

According to the median expression of LINC02126, samples from lung adenocarcinoma patients were divided into high expression and low expression group. In survival analyses, low expression of LINC02126 was significantly related to poor prognosis and increased risk of cancer-related death in lung adenocarcinoma patients. Patients with low LINC02126 expression had a shorter median survival time than those with high LINC02126 expression (Fig. 2A and 2B). The expression level of LINC02126 had a prognostic value for the long-term survival of lung adenocarcinoma patients (Fig. 2C). The AUC for 1, 3 and 5 years were 0.576, 0.567 and 0.607, respectively.

**Fig. 3:**
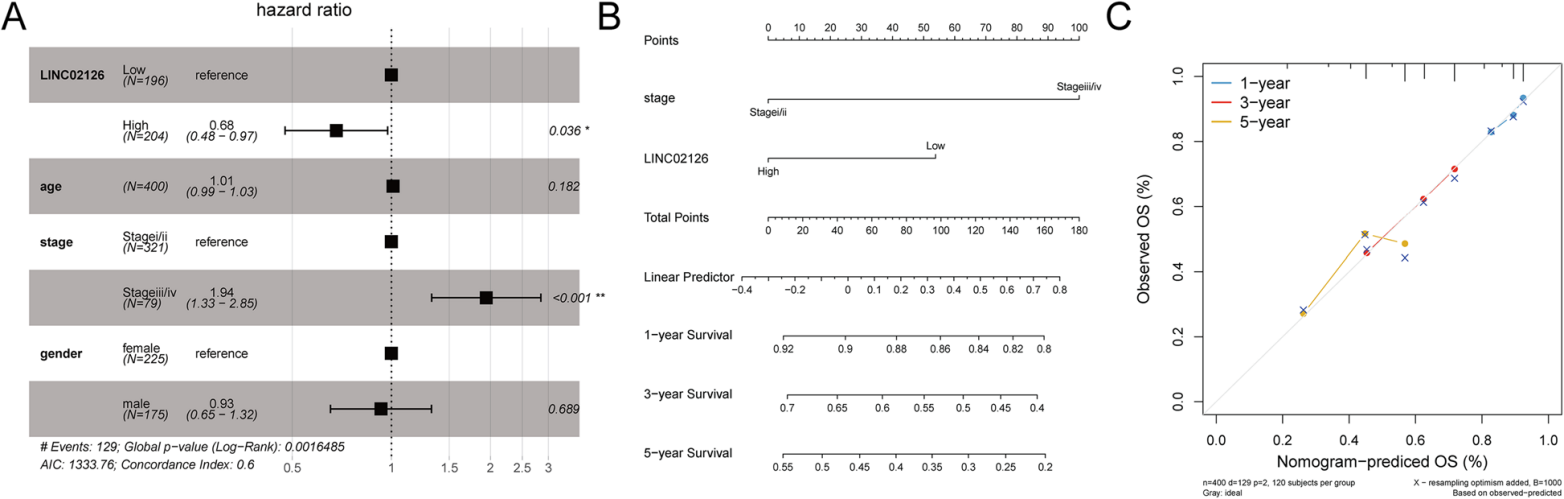
Relationship between LINC02126 and clinical features of lung adenocarcinoma. **A** Multivariate Cox analysis showed that LINC02126 was an independent prognostic factor different from age, stage and grade; **B** The Nomogram for predicting 1, 3 and 5 years survival possibilities; points represent the single score of stage or LINC02126, total points represents the total score of stage and LINC02126; **C** The calibration curve of 1, 3 and 5 years of survival

Age and tumor grade are important clinical information of patients. Therefore, it is necessary to clarify the relationship between tumor risk score and clinical features. Multivariate Cox analysis showed that LINC02126 was an independent prognostic factor different from age, stage and grade (Fig. 3A). LINC02126 and stage, which were independent prognostic indicators in multivariate Cox analysis, were applied to construct a Nomogram to predict the probability of overall survival at 1, 3 and 5 years. As showed in Fig. 3B, each factor was assigned in proportion to its risk contribution to survival. Based on the calibration curves, the Nomogram showed a high accuracy in 1, 3, and 5 years overall survival (Fig. 3C).

### Relationship between LINC02126 and immune cell infiltration

**Fig. 4:**
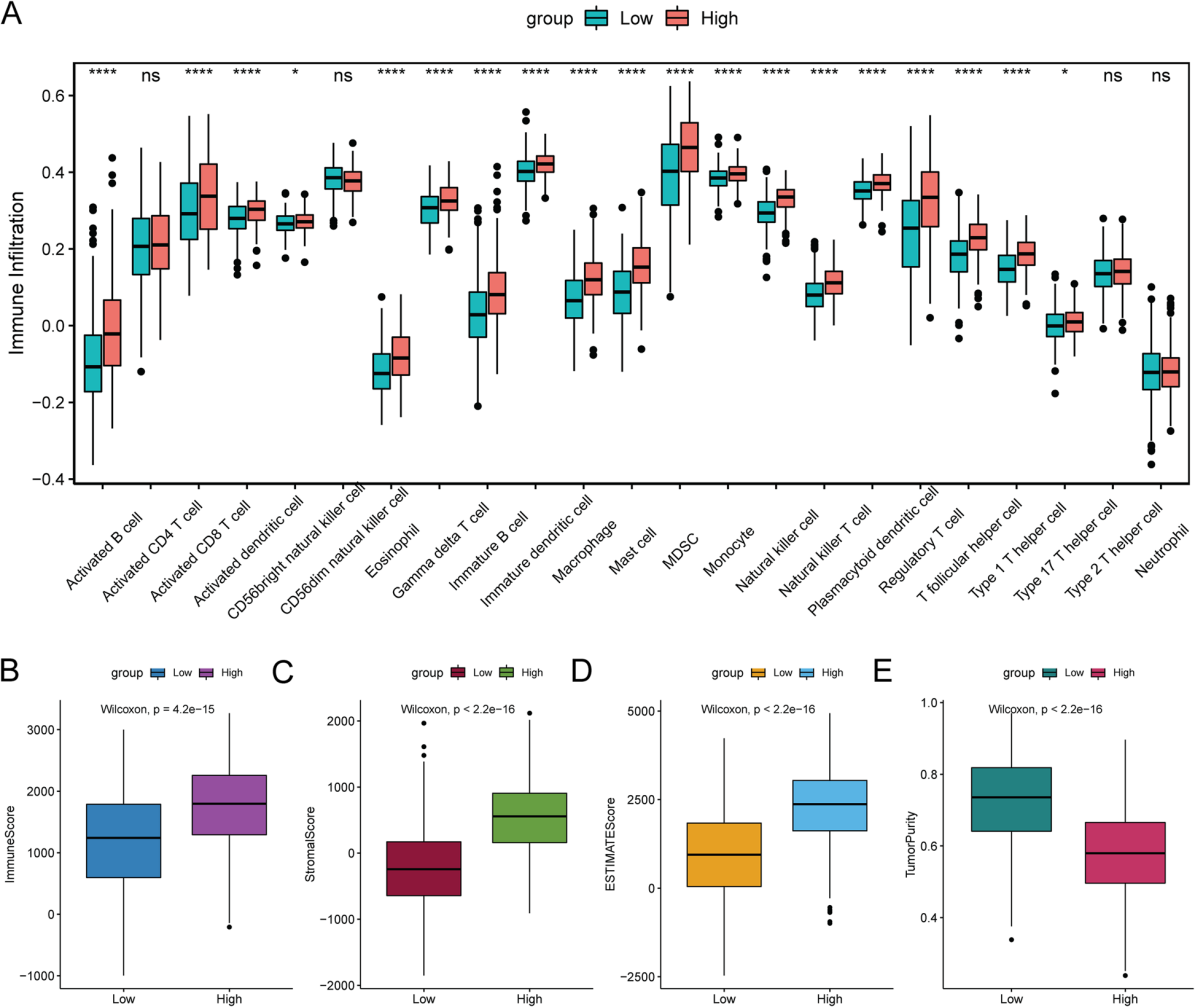
Relationship between high and low LINC02126 expression and immune cell infiltration in lung adenocarcinoma. **A** box plots of the proportion of immune cell infiltration; **B** Differences in immune score; **C** Differences in stroma score; **D** Differences in ESTIMATE score; **E** Differences in tumor purity

To explore the relationship between LINC02126 and TIME, ssGSEA method was used to evaluate the status of 23 kinds of immune cell infiltration in lung adenocarcinoma. The difference in immune cell infiltration between the low and high LINC02126 expression groups was analyzed. The infiltration degree of most immune cells in the low LINC02126 expression group was significantly lower than that in the high LINC02126 expression group, such as activated B cell, immature B cell, natural killer cell, T follicular helper cell and neutrophil (Fig. 4A). Immunological score (Fig. 4B), stroma score (Fig. 4C), and ESTIMATE score (Fig. 4D) was significantly lower in the low LINC02126 expression group than in the high LINC02126 expression group. Tumor purity was significantly higher in the low LINC02126 expression group (Fig. 4E). These results suggest that in the low LINC02126 expression group, the reduced infiltration of immune cells in the TIME could contribute to the poor prognosis of lung adenocarcinoma patients.

### Relationship between LINC02126 and tumor mutation burden

**Fig. 5:**
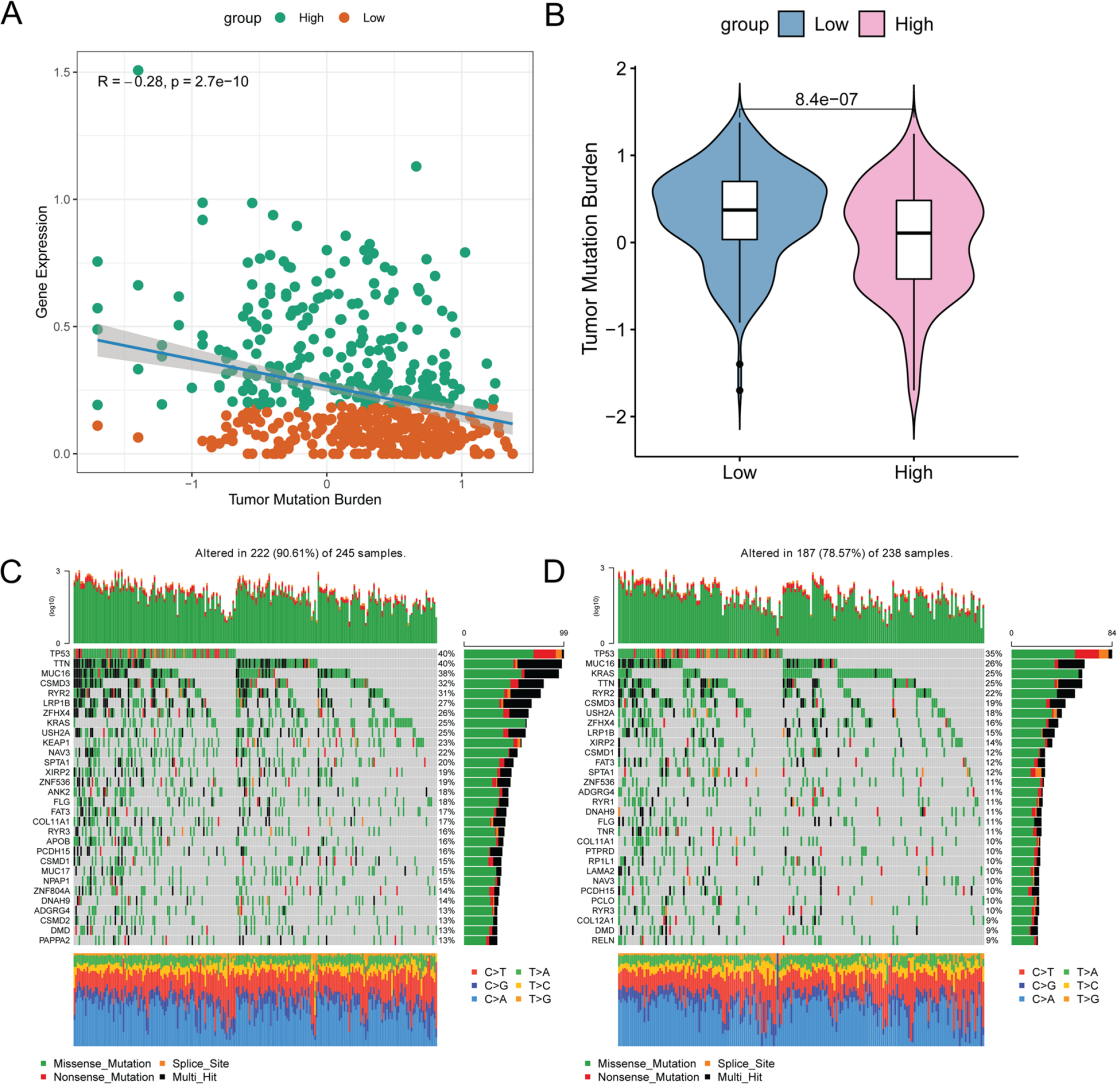
Relationship between high and low LINC02126 expression and tumor mutation burden in lung adenocarcinoma. **A** Correlation linear regression analysis; **B** Differences in tumor mutation burden; **C** Cascade of mutations in the low LINC02126 expression group; **D** Cascade of mutations in the high LINC02126 expression group

Recently, high tumor mutation burden has been identified as a genetic trait related to favorable outcomes of immune checkpoint inhibitor therapy. More and more evidence suggested that tumor mutation burden may determine the individual's response to cancer immunotherapy. It is a meaningful research content to explore the internal relationship between tumor mutation burden and LINC02126. The "MAftools" package in R was utilized to calculate tumor mutation burden score. The correlation analysis was performed between LINC02126 and tumor mutation burden. The result showed that LINC02126 was negatively correlated with tumor mutation burden (Fig. 5A). Tumor mutation burden level of patients with low LINC02126 expression group was significantly higher than that of patients with high LINC02126 expression group (Fig. 5B). To further evaluate the distribution of somatic variation in the driver genes between the low and high LINC02126 expression groups, the top 30 driver genes with the highest change frequency were compared. The frequency of somatic mutation in LINC02126 low expression group (Fig. 5C) was significantly higher than that in LINC02126 high expression group (Fig. 5D). For example, mutation frequency of titin (TTN, 40%), mucin 16, cell surface associated (MUC16, 38%) and CUB and Sushi multiple domains 3 (CSMD3, 32%) was slightly higher in the LINC02126 low expression group compared with that LINC02126 high expression group. This suggested that mutation of these genes may be associated with low expression of LINC02126.

### Functional analysis of genes co-expressed with LINC02126

**Fig. 6:**
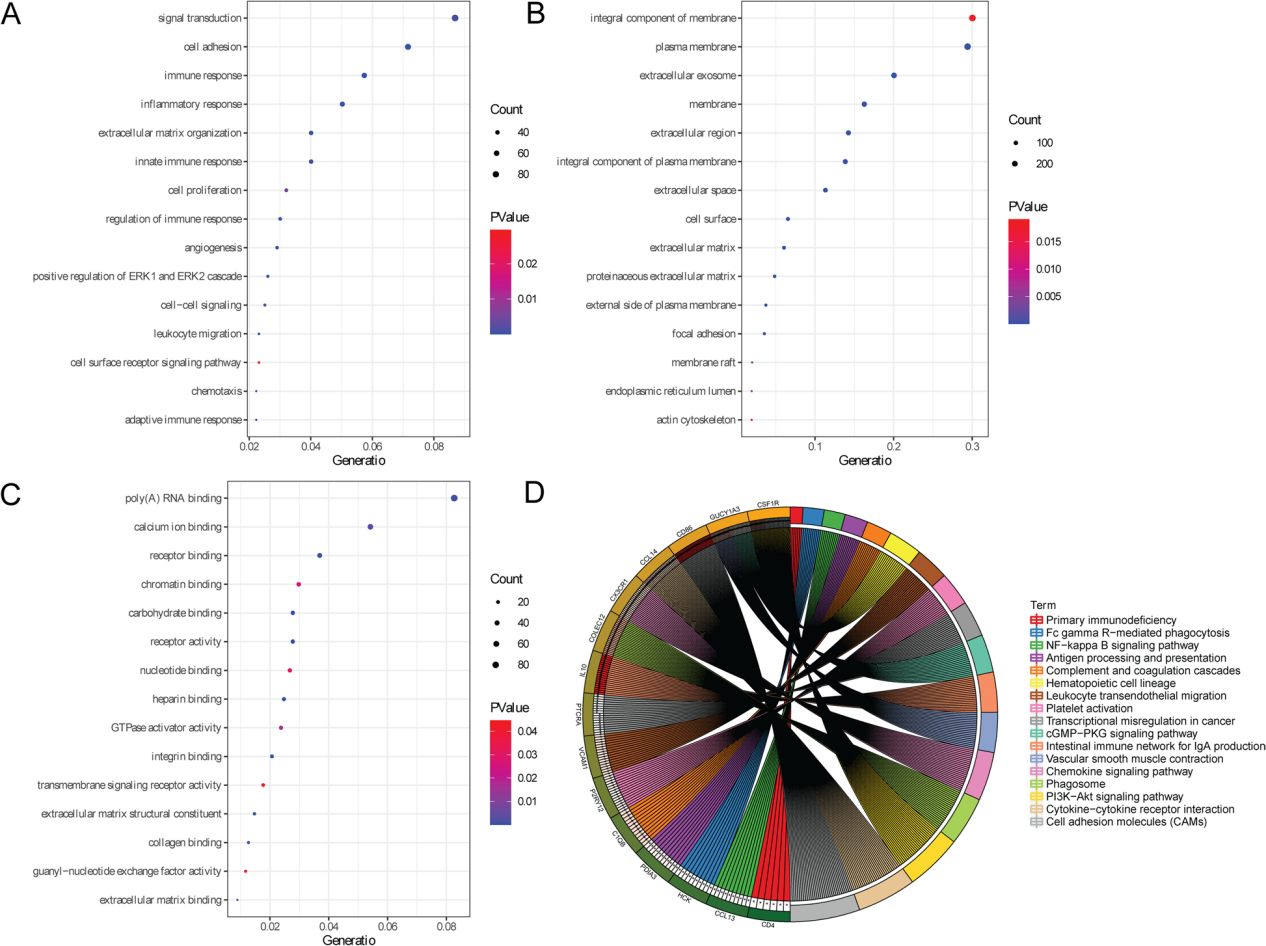
Functional analysis of genes co-expressed with LINC02126 in lung adenocarcinoma **A** Biological process; **B** Cytological component; **C** Molecular function; **D** KEGG analysis

It is well known that lncRNAs are non-coding RNAs that participate in regulating the expression level of genes in the post-transcriptional level. A total of 990 genes were co-expressed with LINC02126. Among which, 189 genes were negatively associated with LINC02126, while 801 genes were positively associated with LINC02126. Some co-expressed genes that positively correlated with LINC02126 were identified (supplementary Table 1), such as decorin (DCN), cytochrome c oxidase subunit 7A1 (COX7A1), placenta associated 9 (PLAC9), lumican (LUM) and microfibril associated protein 4 (MFAP4). DAVID was used to assess the function of these co-expressed genes with LINC02126. GO analysis showed that signal transduction, integral component of membrane and poly(A) RNA binding was the most significantly enriched biological process, cytological component and molecular function, respectively (Fig. 6A, 6B and 6C). Some significantly enriched signaling pathways were identified in the KEGG analysis, such as cell adhesion molecules (CAMs), intestinal immune network for IgA production, phagosome, vascular smooth muscle contraction, hematopoietic cell lineage, cytokine-cytokine receptor interaction and chemokine signaling pathway.

### Functional analysis of DEGs in different LINC02126 expression groups

**Fig. 7:**
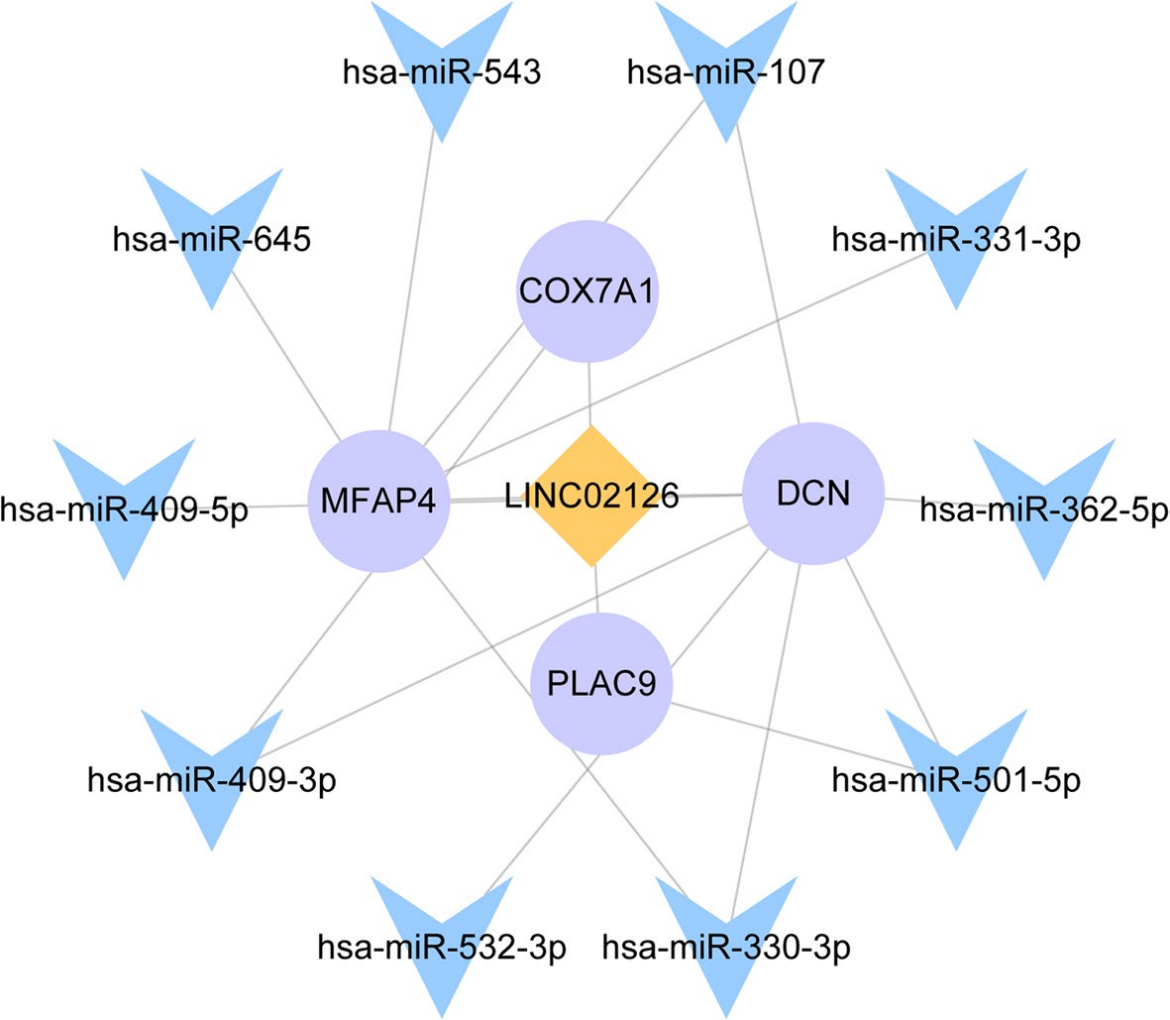
The regulatory relationship between LINC02126, 10 miRNAs and DCN, COX7A1, PLAC9, and MFAP4 in lung adenocarcinoma

**Fig. 8:**
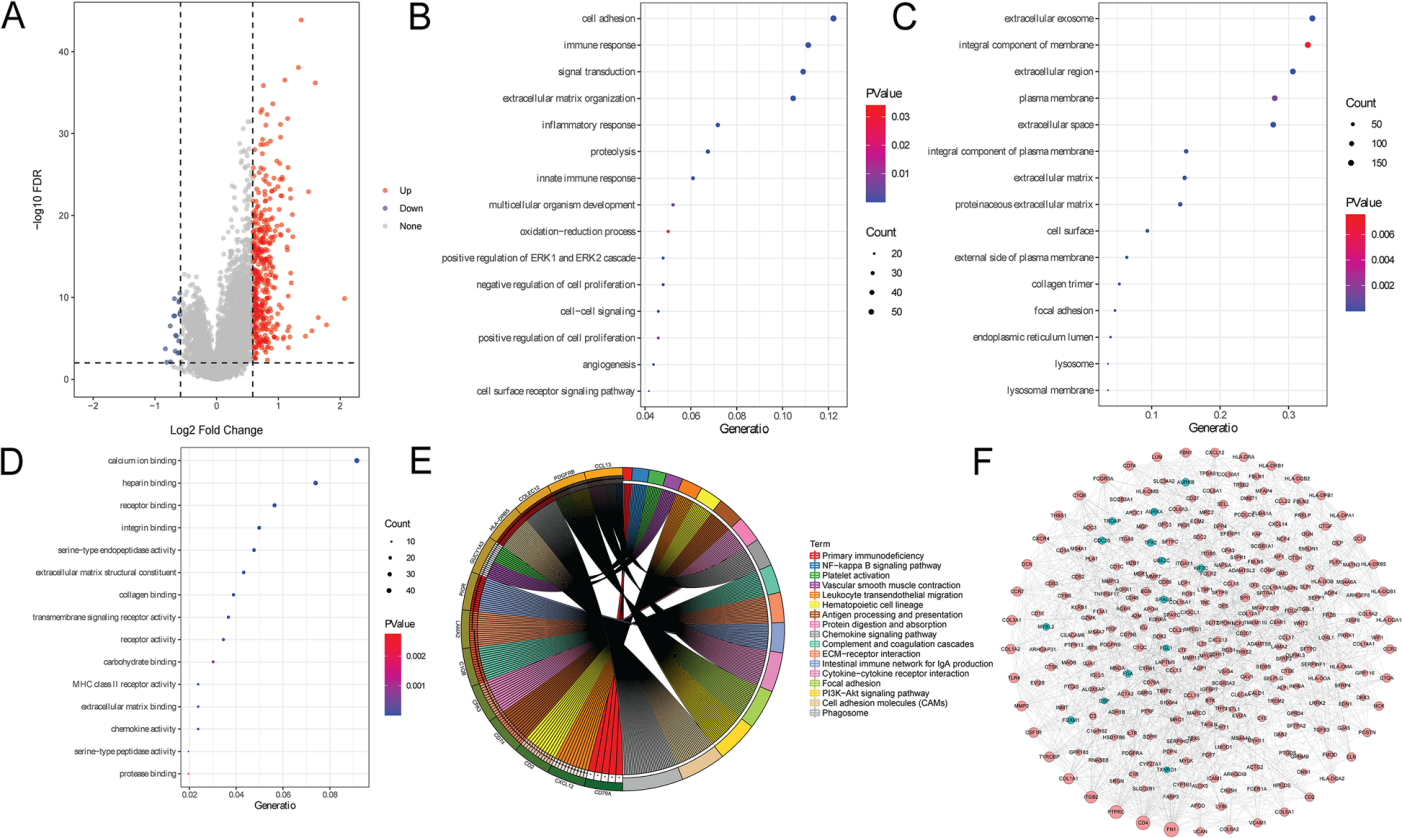
Functional analysis of DEGs in different LINC02126 expression groups in lung adenocarcinoma. **A** Volcanic map of DEGs; **B** Biological process; **C** Cytological component; **D** Molecular function; **E** KEGG analysis; **F **PPI network

Based on the median expression of LINC02126, a total of 458 (19 up-regulated and 439 down-regulated) DEGs were identified between low and high LINC02126 expression groups. There were 297 common genes between DEGs and co-expressed genes, such as DCN (up-regulation), COX7A1 (up-regulation), PLAC9 (up-regulation), LUM (up-regulation) and MFAP4 (up-regulation) (supplementary Table 2). The heat map of common genes is shown in Supplementary Fig. 1. Additionally, the regulatory relationship between LINC02126, miRNAs and DCN, COX7A1, PLAC9, and MFAP4 was analyzed. From the miRWalk database, 1547 miRNAs were found to be interacted with the above 4 genes. In addition, 216 differentially expressed miRNA were identified in lung adenocarcinoma from GSE74190 dataset. After taking the intersection, 10 miRNA that negatively regulated the above 4 genes were identified, including hsa-miR-107, hsa-miR-362-5p, hsa-miR-409-5p, hsa-miR-532-3p, hsa-miR-330-3p, hsa-miR-501-5p, hsa-miR-409-3p, hsa-miR-645, hsa-miR-543, and hsa-miR-331-3p. The regulatory network between LINC02126, 10 miRNA and the above 4 genes was constructed (Fig. 7). The volcanic diagram of 458 DEGs is shown in Fig. 8A.

In order to explore the potential biological function of DEGs in different LINC02126 expression groups, GO and KEGG was performed. GO analysis of these DEGs showed that cell adhesion, extracellular exosome and calcium ion binding were the most significantly enriched biological process, cytological component and molecular function, respectively (Fig. 8B, 8C and 8D). It is noted that some enriched signaling pathways of genes co-expressed with LINC02126 were also found in functional analysis of DEGs in different LINC02126 expression groups, including cell adhesion molecules (CAMs), intestinal immune network for IgA production, phagosome, vascular smooth muscle contraction, hematopoietic cell lineage, cytokine-cytokine receptor interaction and chemokine signaling pathway (Fig. 8E). These KEGG pathways have been obtained the copyright by Kanehisa laboratories [24]. In the PPI network, complex interaction between these DEGs was observed (Fig. 8F).

## Discussion

Up to now, there are no reports about LINC02126 in any disease. In this study, the expression of LINC02126 was significantly lower in tumors tissues of lung adenocarcinoma than that in adjacent tissues. In addition, significant expression differences of LINC02126 were found in T staging (low expression in T3/4), M staging (low expression in M1), and stage (low expression in iii/iv). This suggested that low expression of LINC02126 was associated with disease progression. Interestingly, low expression of LINC02126 was significant in male patients than that in female patients. In lung cancer, the gender-associated difference in clinical outcome is confirmed in several studies [25–28]. In lung adenocarcinoma, male tumors harbor higher burden of genetic alterations than female counterparts, and greater burden of genetic alterations is related to poor clinical outcomes [29]. In previous study in Asia, female patients showed a survival advantage than male [30]. This indicated that gender may be identified as a prognostic factor for survival in patients with low expression of LINC02126. Moreover, we found that LINC02126 had a potential diagnostic value (with AUC of 0.781) for lung adenocarcinoma. In survival analyses, low expression of LINC02126 was significantly related to poor prognosis and increased risk of cancer-related death in lung adenocarcinoma patients. It is worth mentioning that LINC02126 was an independent prognostic factor different from age, stage and grade. Our results suggested that LINC02126 expression was significantly abnormal in lung adenocarcinoma and had a high accuracy in the disease diagnosis and prognosis, which could be used as a potential diagnostic and prognostic biomarker of the disease.

Immunotherapy has been identified as an indispensable method for cancer treatment [31]. Immunotherapy is an emerging novel treatment for several cancers, especially lung adenocarcinoma. Moreover, multitudes of investigations have indicated that lncRNAs play non-negligible roles in cancer immunity [32, 33]. Based on relationship analysis between LINC02126 and immune cell infiltration, we found that the infiltration degree of most immune cells in the low LINC02126 expression group was significantly lower, such as activated B cell, immature B cell, natural killer cell, T follicular helper cell and neutrophil. Moreover, immunological score, stroma score, and ESTIMATE score was significantly lower in the low LINC02126 expression group than in the high LINC02126 expression group. B cells positively modulate immune responses and inflammation to promote T-cell activation and proliferation. Tumor infiltrating B cells appear in every stage of lung cancer and play an important role in shaping tumor progression. In non-small cell lung cancer, the infiltration of B cells is associated with the good prognosis [34]. Some immune cells has been found in lung adenocarcinoma, such as B cells naïve and B cells memory [35]. It is suggested that tumor-infiltrating B cells acts as the clinical factor in anti-PD-L1 immunotherapy for lung adenocarcinoma [36]. Natural killer cell can exert natural cytotoxicity against cancer cells and inhibit metastasis to different tissues [37]. Natural killer cell-mediated cytotoxicity is a key anti-tumor mechanism [38, 39]. In higher-risk patients with lung adenocarcinoma, a significant depletion of activated natural killer cells was observed, indicating the transformation of innate immunity in TIME from activating to suppressive status [40]. T follicular helper cells play important roles in the development of immunity. In lung cancer tissues, T follicular helper cells can secrete the chemokine to attract B cells into the tumor tissue [41]. Neutrophil autophagy is closely associated with neutrophil immune activity and cytokine secretion [42, 43]. In tumors, neutrophils often predict worsened outcomes. Low levels of neutrophils are related to poor prognosis in patients with lung adenocarcinoma [44]. Our result indicated that low expression of LINC02126 was related to the reduced infiltration of immune cells in the TIME, which may contribute to the poor prognosis of lung adenocarcinoma patients. It is assumed that LINC02126 may be considered as a potential immunotherapy target for lung adenocarcinoma.

According to the relationship analysis between LINC02126 and tumor mutation burden, we found that LINC02126 was negatively correlated with tumor mutation burden. Tumor mutation burden level of patients with low LINC02126 expression group was significantly higher than that of patients with high LINC02126 expression group. Mutation frequency of TTN, MUC16 and CSMD3 was slightly higher in the LINC02126 low expression group compared with that in LINC02126 high expression group. Somatic mutations in TTN are frequently found in some cancer types and reflect the status of the tumor mutation burden [45, 46]. In lung adenocarcinoma, the mutation frequency of TTN is significantly associated with the response rate to immune checkpoint blockades [47]. For lung adenocarcinoma patients, MUC16 is found to be one of the most commonly mutated genes in predicted neo-antigens [48]. In lung adenocarcinoma, somatic mutations of CSMD3 are up-regulated in the *TP53* gene mutation status [49]. Our result suggested that mutations of TTN, MUC16 and CSMD3 may be associated with low expression of LINC02126 in lung adenocarcinoma.

In addition, we found that some DEGs were also co-expressed with LINC02126, such as up-regulated DCN, COX7A1, PLAC9, LUM and MFAP4. Hypomethylated and highly expression of DCN are related to poor prognosis in lung adenocarcinoma [50]. COX7A1, PLAC9 and LUM are up-regulated in lung adenocarcinoma [51, 52]. MFAP4 is associated with elastogenesis in lung [53]. In lung adenocarcinoma, has-miR-147b promotes cells malignant aggressiveness by targeting MFAP4 [54]. In addition, we found that some miRNAs were involved in the regulatory network between LINC02126, DCN, COX7A1, PLAC9, and MFAP4, such as hsa-miR-107, hsa-miR-532-3p, hsa-miR-330-3p, hsa-miR-501-5p, hsa-miR-409-3p, hsa-miR-543, and hsa-miR-331-3p. Hsa-miR-107 is involved in cell proliferation of to non-small cell lung cancer [55]. It is suggested that hsa-miR-532-3p may serve as a prognostic marker for lung adenocarcinoma [56]. It has been demonstrated that hsa-miR-330-3p promote invasion and metastasis of non-small cell lung cancer. Hsa-miR-501-5p is associated with paclitaxel-resistant non-small cell lung cancer cells [57]. Hsa-miR-409-3p is involved in lymph node metastasis of LC [58]. Hsa-miR-543 promotes tumorigenesis and angiogenesis in non-small cell lung cancer [59]. Hsa-miR-331-3p is associated with prognostic value in lung adenocarcinoma [60]. This suggested that LINC02126 may play important roles in the development of lung adenocarcinoma by regulating the expression of DCN, COX7A1, PLAC9, LUM and MFAP4.

In addition, we found that those DEGs that co-expressed with LINC02126 were significantly enriched in some pathways, including cell adhesion molecules (CAMs), intestinal immune network for IgA production, phagosome, vascular smooth muscle contraction, hematopoietic cell lineage, cytokine-cytokine receptor interaction and chemokine. Decreased cell adhesion in lung cancer cells is related to cancer spread through vessels or alveolar space [61–63]. In lung adenocarcinoma, carcinoembryonic antigen-related cell adhesion molecules can be considered as surrogate markers for epidermal growth factor receptor (EGFR) inhibitor sensitivity [64]. The function of the intestinal immune network for IgA production is to generate non-inflammatory IgA antibodies, which are indicators of immune function. The immune-associated pathway of the intestinal immune network for IgA production is found in lung adenocarcinoma patients [65]. Phagosome is an important immune and inflammatory pathway in lung adenocarcinoma patients [65]. In lung cancer, the vascular smooth muscle contraction pathway is negatively regulated during tumorigenesis [66]. The hematopoietic cell lineage is involved in cell-type-specific crosstalk [67]. The loss of some hematopoietic cells is related to aggressive lung adenocarcinoma [68]. Cytokines, secreted glycoproteins, functions as intercellular mediators and promote cellular proliferation and apoptosis [69]. In addition, cytokines promotes the recruitment of immune-suppressive cells, leading to tumor metastasis [70]. Cytokine-cytokine receptor interaction signaling pathway plays significant roles in the prognosis of lung adenocarcinoma [71]. Chemokine receptors and its ligands are related to tumor progression and metastasis [72, 73]. The aberrant expression of chemokine receptors has been related to poor prognosis in lung cancer [74, 75]. This indicated that above signaling pathways might be associated with LINC02126-mediated tumorigenesis in lung adenocarcinoma.

## Conclusions

In summary, LINC02126 may be considered as the independent diagnostic and prognostic factor and reflect the overall intensity of the immune response in the lung adenocarcinoma TIME. However, there are limitations to our study. Potential biological mechanism of identified co-expressed genes with LINC02126 and related signaling pathways is not investigated. In vitro cell experiment is needed to further explore the function of LINC02126.

## Supplementary Information

**Additional file 1: Supplementary Figure 1.** The heat map of common genes between DEGs and co-expressed genes

**Additional file 2**.

**Additional file 3.**

## Data Availability

RNA sequencing data and clinical information were downloaded from the UCSC Xena (https://gdc.xenahubs.net).

## Abbreviations

AUC: Area under the curve
CAMs: Cell adhesion molecules
CCAT2: Colon cancer-associated transcript 2
COX7A1: Cytochrome c oxidase subunit 7A1
DCN: Decorin
DEGs: Differentially expressed genes
EGFR: Epidermal growth factor receptor
FDR: False discovery rate
FC: Fold change
GTEx: Genotype-tissue expression
lncRNAs: Long non-coding RNAs
LUM: Lumican
MALAT1: Metastasis-associated lung adenocarcinoma transcript 1
MFAP4: Microfibril associated protein 4
PLAC9: Placenta associated 9
PCA3: Prostate cancer antigen 3
PPI: Protein–protein interaction
ROC: Receiver operating characteristic
ssGSEA: Single sample gene set enrichment analysis
TCGA: The Cancer Genome Atlas
TIME: Tumor immune microenvironment
UCSC: University of California, Santa Cruz
