# Supplementary figures and images for: LINC02126 is a potential diagnostic, prognostic and immunotherapeutic target for lung adenocarcinoma
Source: BMC Pulm Med. 2022 Nov 10;22:412. doi: 10.1186/s12890-022-02215-4 (PMC9650865; doi:10.1186/s12890-022-02215-4)

Supplementary Figure 1 The heat map of common genes between DEGs and co-expressed genes


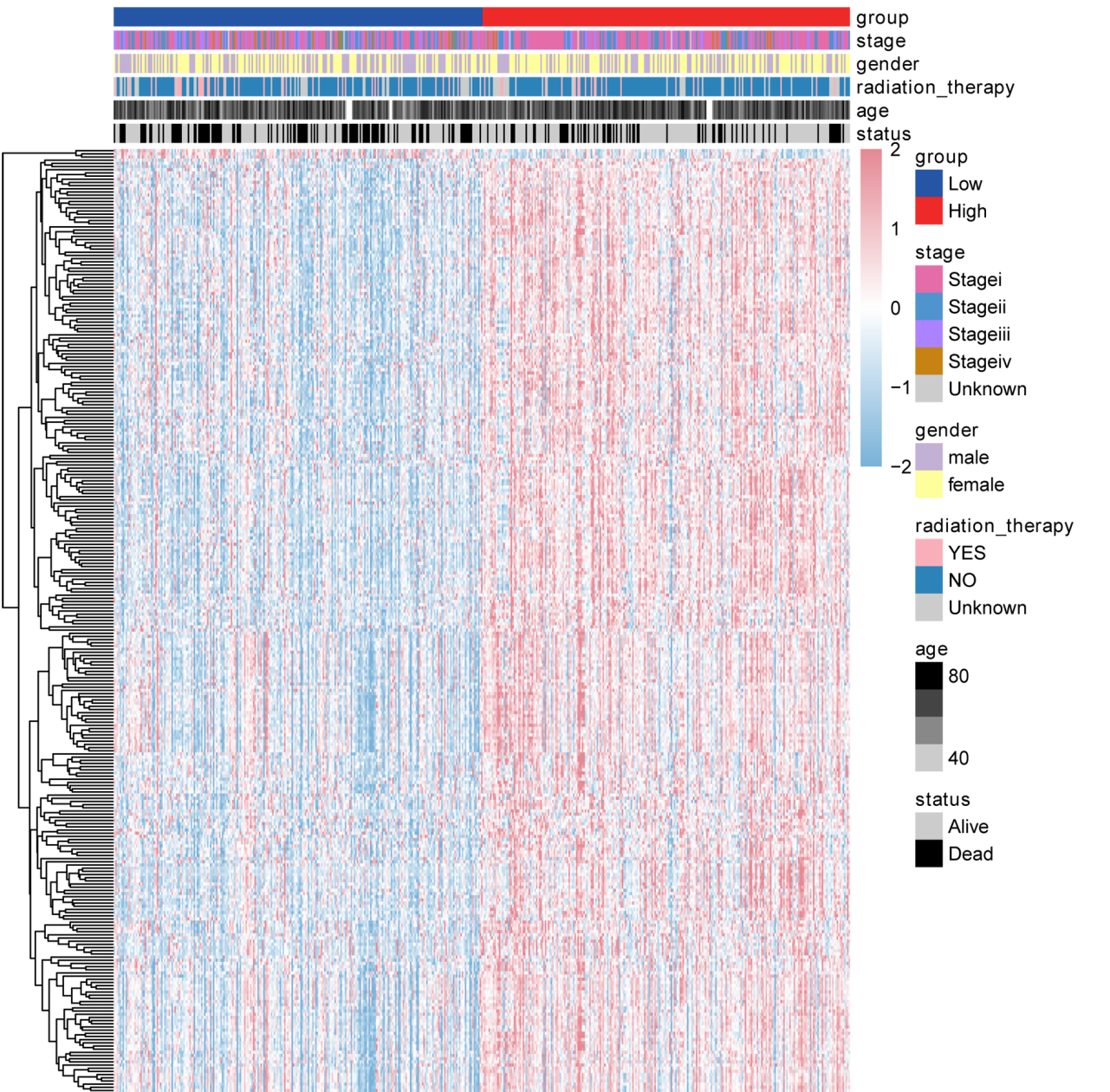

Supplement: Supplementary file 1 — Additional file 1: Supplementary Figure 1. The heat map of common genes between DEGs and co-expressed genes [file 12890_2022_2215_MOESM1_ESM.docx]
